# Early detection of doxorubicin-induced cardiotoxicity in rats by its cardiac metabolic signature assessed with hyperpolarized MRI

**DOI:** 10.1038/s42003-020-01440-z

**Published:** 2020-11-19

**Authors:** Kerstin N. Timm, Charith Perera, Vicky Ball, John A. Henry, Jack J. Miller, Matthew Kerr, James A. West, Eshita Sharma, John Broxholme, Angela Logan, Dragana Savic, Michael S. Dodd, Julian L. Griffin, Michael P. Murphy, Lisa C. Heather, Damian J. Tyler

**Affiliations:** 1 Department of Physiology Anatomy and Genetics, University of Oxford, Sherrington Building, Parks Road, Oxford, OX1 3PT UK; 2 Department of Biochemistry, University of Cambridge, Tennis Court Road, Cambridge, CB2 1QW UK; 3 Wellcome Centre for Human Genetics, University of Oxford, Roosevelt Dr, Headington, Oxford, OX3 7BN UK; 4 MRC Mitochondrial Biology Unit, University of Cambridge, The Keith Peters Building, Cambridge Biomedical Campus, Hills Road, Cambridge, CB2 0XY UK; 5 Oxford Centre for Clinical Magnetic Resonance Research, John Radcliffe Hospital, Headington, Oxford, OX3 9DU UK

**Keywords:** Metabolomics, Heart failure, Translational research, Preclinical research, Predictive markers

## Abstract

Doxorubicin (DOX) is a widely used chemotherapeutic agent that can cause serious cardiotoxic side effects culminating in congestive heart failure (HF). There are currently no clinical imaging techniques or biomarkers available to detect DOX-cardiotoxicity before functional decline. Mitochondrial dysfunction is thought to be a key factor driving functional decline, though real-time metabolic fluxes have never been assessed in DOX-cardiotoxicity. Hyperpolarized magnetic resonance imaging (MRI) can assess real-time metabolic fluxes in vivo. Here we show that cardiac functional decline in a clinically relevant rat-model of DOX-HF is preceded by a change in oxidative mitochondrial carbohydrate metabolism, measured by hyperpolarized MRI. The decreased metabolic fluxes were predominantly due to mitochondrial loss and additional mitochondrial dysfunction, and not, as widely assumed hitherto, to oxidative stress. Since hyperpolarized MRI has been successfully translated into clinical trials this opens up the potential to test cancer patients receiving DOX for early signs of cardiotoxicity.

## Abstract (web summary)

Using hyperpolarized 13C magnetic resonance imaging (MRI), Timm et al. show that a change in oxidative mitochondrial carbohydrate metabolism precedes cardiac decline in a rat model of heart failure induced by doxorubicin (DOX), a widely used chemotherapeutic agent. This study suggests the possibility of using hyperpolarized MRI to test cancer patients receiving DOX for early signs of cardiotoxicity.

## Introduction

Doxorubicin (DOX) is a commonly used chemotherapeutic agent for the treatment of adult and pediatric cancers, such as breast cancer and lymphoma. DOX has greatly improved cancer survival rates, however, even when limiting life-time doses to <400 mg m^−2^, the incidence of developing heart failure (HF)^1^ as a result of DOX treatment remains at ~5%^2^. Therefore, the early detection and targeted treatment of patients at risk from cardiotoxicity is paramount to reducing the incidence of life-limiting DOX-HF in cancer survivors. Unfortunately, there are currently no clinical imaging techniques or biomarkers available to detect DOX-cardiotoxicity before the onset of functional decline, and there are no specific treatments to prevent the onset of DOX-HF^3^.

Numerous molecular mechanisms have been proposed for DOX-cardiotoxicity, all culminating in cardiomyocyte death^4^. These mechanisms include alterations to autophagy^5^, mitophagy^6^ and mitochondrial dysfunction^7^. Production of mitochondrial reactive oxygen species (ROS) by iron-dependent and independent mechanisms is thought to be upstream of the above mechanisms^8^, but these observations stem solely from preclinical models and are challenging to assess in patients in the clinic. Furthermore, iron-chelating agents, such as dexrazoxane, which confer some level of cardioprotection in breast cancer patients^9^, have not shown the expected efficacy should oxidative stress truly be at the core of DOX-HF^8^. Furthermore, any cardioprotective effect of dexrazoxane has recently been attributed to its interaction with topoisomerase 2β (TOP2β) rather than through its iron-binding capacity^10^. It is proposed that dexrazoxane could prevent DOX binding to TOP2β and inhibit the generation of DOX-induced DNA double strand breaks that initiate apoptosis. In addition, dexrazoxane is contra-indicated in children because it can lead to cancer formation^11^, further emphasizing the need for targeted therapies to prevent DOX-HF. Studies in mice have shown cardioprotection when Top2β^12^, BNIP3^13^ (a protein involved in mitophagy), or iNOS^14^ (inducible nitric oxide synthase) are knocked out. Given that these are examples of very different and distinct pathways (DNA-damage, mitophagy, nitrosative/oxidative stress) it is clear that, either (i) the primary target of DOX has not yet been identified, (ii) that DOX acts on a range of cellular targets simultaneously, or (iii) that different model systems and treatment regimens introduce phenotypes with underlying pathology that do not necessarily mimic DOX-HF in patients.

Cardiac energetics are thought to play a key role in the development of DOX-HF^15^ and indeed in heart failure in general^16^. Noninvasively measuring cardiac metabolism in vivo could allow the early detection of the cardiotoxic effects of DOX on metabolism in patients, facilitating prophylactic treatment before the onset of irreversible functional decline. Radiolabeled analogs of glucose and fatty acids have previously been used with positron emission tomography/computed tomography (PET/CT) to detect changes in substrate uptake in the DOX-treated heart, which precede functional changes in rats^17^. However, these measurements pose a potential health-risk to the patient due to the radioactive dose from both the tracer and the CT image acquisition for anatomical localization, although this is mainly a concern in pediatric patients. Furthermore, PET only measures substrate accumulation or dynamic uptake while true measurements of downstream real-time metabolism have not previously been possible. Magnetic resonance (MR) imaging and spectroscopy in turn may offer a safe and noninvasive measure of cardiac structure, function and substrate utilization in vivo in one imaging session. Carbon-13 (^13^C) MR spectroscopy (MRS) is particularly well suited to studying metabolism, due to the ubiquitous presence of carbon in metabolites and the large range of relevant chemical shifts in ^13^C MR spectra. Until recently the low natural abundance of ^13^C nuclei had prevented real-time measurements of metabolic fluxes in vivo. However, over the last decade, hyperpolarized MRS by dissolution dynamic nuclear polarization of ^13^C-labeled substrates^18^ has revolutionized cardiac metabolic flux measurements in preclinical models^19^ and in the human heart^20,21^. For example, increased lactate labeling from hyperpolarized [1-^13^C]pyruvate was recently shown to be a marker of innate immune cell driven inflammation in a rodent model of myocardial infarction^22^. Furthermore, by selective labeling of pyruvate as either [1-^13^C] or [2-^13^C], both cytosolic anaerobic and mitochondrial oxidative carbohydrate metabolism can be measured in real-time^23,24^.

Here we show that hyperpolarized [1-^13^C]pyruvate allows early detection of reduced mitochondrial oxidative carbohydrate metabolism in a clinically-relevant rat model of DOX-HF. The decrease in mitochondrial oxidative carbohydrate metabolism was indicative of mitochondrial loss and dysfunction, which was not caused by oxidative stress.

## Results and discussion

### Doxorubicin treatment in rats leads to cardiac dysfunction

**Fig. 1:**
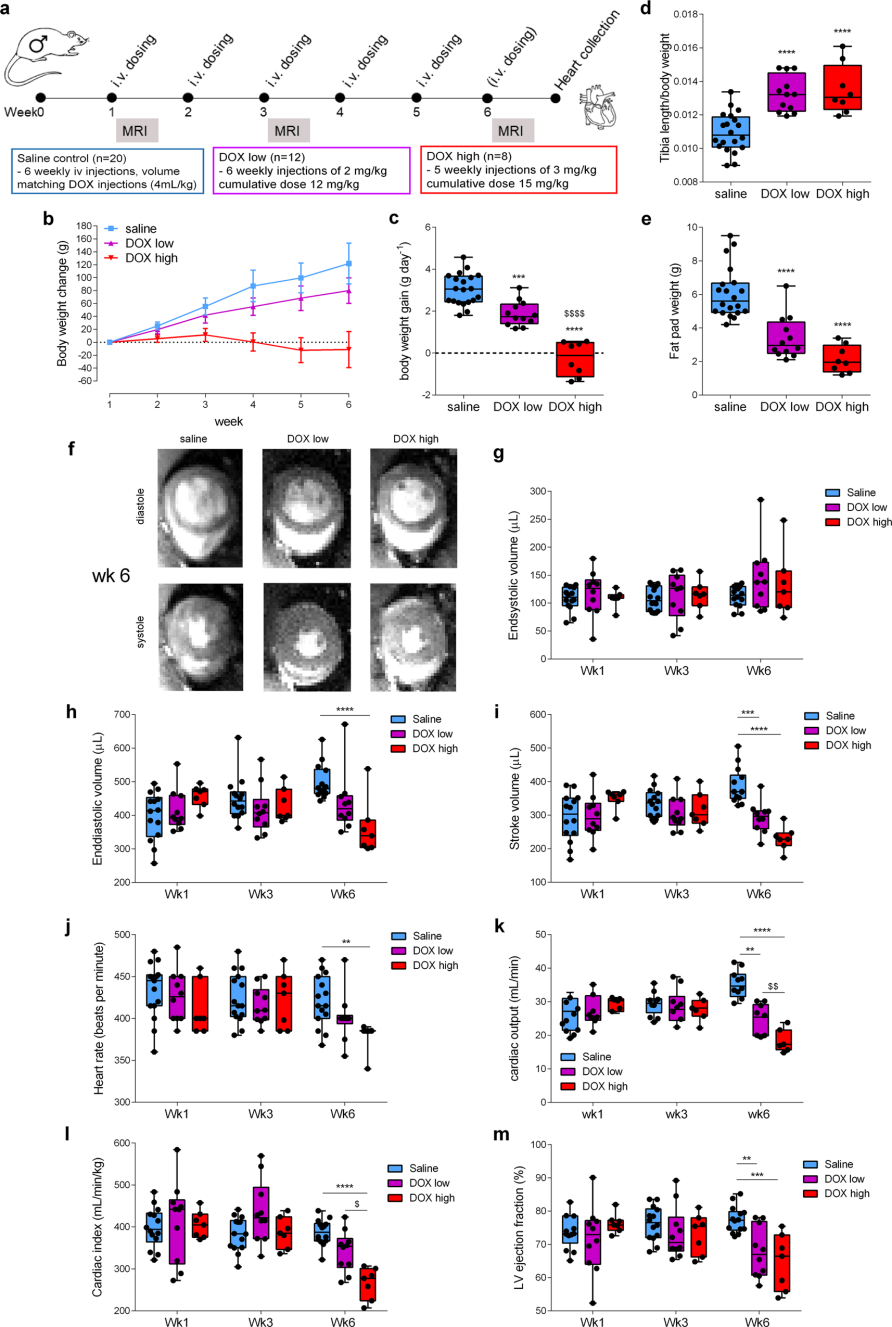
A clinically-relevant rat model of doxorubicin-induced heart failure. **a** Outline of the study. Male Wistar rats were treated intravenously (i.v.) with weekly injections of either sterile saline or doxorubicin (DOX) in three groups: saline, DOX low, DOX high. Magnetic resonance imaging (MRI) at weeks 1, 3, and 6 consisted of cardiac function assessed with CINE MRI, and cardiac metabolic flux measurements with hyperpolarized MRI. **b** Rat growth curves (mean ± SEM) and **c** average rat body weight gain per day throughout the 6-week study. After the week 6 imaging timepoint rats were sacrificed for tissue collection. Postmortem measurements of tibia length to assess **d** tibia length:body weight ratio and postmortem **e** epididymal fat pad weights. **f** Representative CINE MR images of maximum diastole (top panel) and systole (bottom panel) in male Wistar rats from saline control, DOX low, and DOX high groups at the final timepoint (week 6). **g** Left ventricular end-systolic volume **h** end-diastolic volume **i** stroke volume **j** heart rate **k** cardiac output **l** cardiac index and **m** left ventricular (LV) ejection fraction, in rats from all three groups at weeks 1, 3, and 6. Box and whisker plots ranging from min to max value with the median indicated by horizontal line. Some graphs do not start at *y* = 0 to allow for better visualization of the data spread. Statistical comparison by one-way ANOVA (**c**–**e**), or two-way ANOVA (**g**–**m**) with Tukey’s HSD correction method for multiple comparisons. **P < 0.01, ***P < 0.001, ****P < 0.0001 compared to saline control group. ^$^Statistically significant difference between DOX high and DOX low group. Source data are provided in Supplementary Data 2.

Male Wistar rats were treated either for six consecutive weeks with sterile saline, with a low dose of 2 mg kg^−1^ DOX (cumulative dose 12 mg kg^−1^) or treated for five consecutive weeks with a high dose of 3 mg kg^−1^ DOX (cumulative dose 15 mg kg^−1^) (Fig. 1a). Similar i.v. doses as used in this study have previously been shown to lead to cardiotoxicity in rats^25,26^. We chose male Wistar rats as mitochondrial energy metabolism is thought to be affected more severely in male rather than female rats^26^. We first assessed the general effects of DOX on the rats by monitoring body weight gain throughout the study, and measuring tibia length and epididymal fat pad weight post mortem at week 6 (Fig. 1b–e). Rats in the low dose DOX group gained weight more slowly than rats in the saline control group. In the high dose DOX group, decreased weight gain was followed by weight loss starting from week 3 (Fig. 1b). Overall, both DOX groups had a significantly reduced average weight gain per day when compared to the saline control group (Fig. 1c). This reduced body weight gain was due to a decrease in body fat deposition and, potentially, loss of lean mass as opposed to a general growth retardation, as evidenced by a significantly increased tibia length:body weight ratio and decreased fat pad weight post mortem in both DOX groups compared to the saline control group (Fig. 1d, e). High dose DOX also led to a significant increase in plasma cardiac troponin I (cTNI) and lactate dehydrogenase (LDH) levels at week 6 (Fig. S1a, b). Overall, DOX treatment in rats led to a dose-dependent effect on body weight gain and an increase in plasma cardiac cellular damage markers indicative of cardiotoxicity.

We next assessed cardiac function in DOX-treated rats by performing CINE MR imaging at weeks 1, 3, and 6 of the study (Fig. 1f–m). Cardiac left ventricular (LV) end-systolic volume was not altered (Fig. 1g) while LV end-diastolic volume was significantly decreased in the DOX high group at week 6 (Fig. 1h). LV stroke volume (SV) was significantly decreased in both DOX-treated groups at week 6 (Fig. 1i). Heart rate was decreased at week 6 only in the DOX high group (Fig. 1j), while cardiac output was significantly decreased in both DOX groups at week 6, and there was a significant difference between the DOX low and DOX high groups (Fig. 1k). After adjusting for body weight, cardiac index was only significantly reduced in the DOX high group, both compared to the saline control group and to the DOX low group (Fig. 1l). LV ejection fraction, the most clinically relevant measure of cardiac function, was reduced at week 6 in both the low and the high dose DOX groups (Fig. 1m).

Overall, we established an in vivo rat model of DOX-cardiotoxicity, using doses and administration routes that are relevant to the clinical setting and leading to a dose-dependent reduction in cardiac function indicative of heart failure. In patients, DOX-induced cardiotoxicity usually presents first with early diastolic dysfunction, followed by systolic dysfunction^27^, and the decreased end-diastolic volume in the rats in this study may indicate a similar pattern. Thus, the rat model of DOX-HF presented here closely mimics the pathology seen in patients and therefore allows us to assess early markers of the development of DOX-HF.

### Cardiac mitochondrial carbohydrate metabolism is impaired in DOX-treated rats

**Fig. 2:**
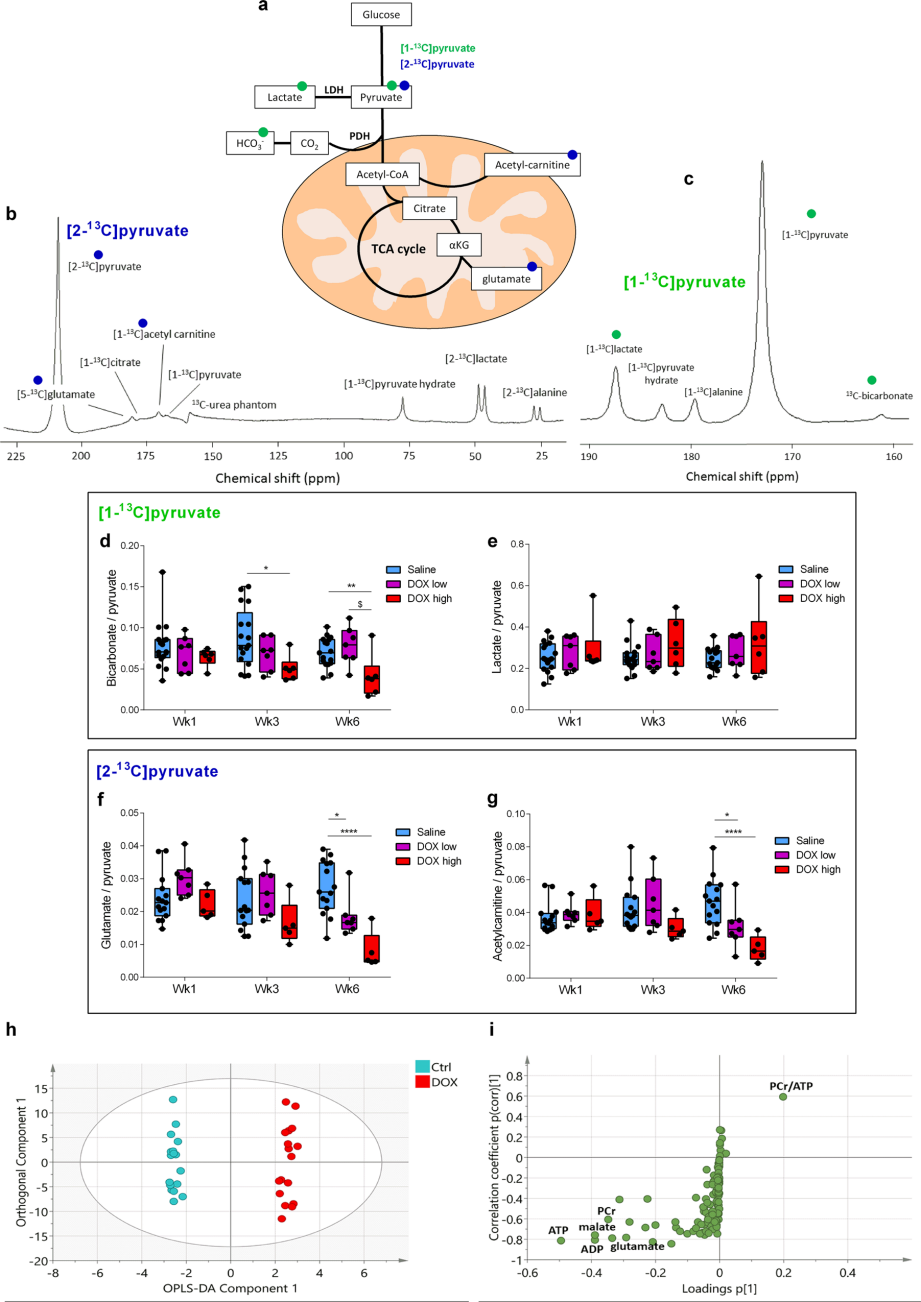
Effects of doxorubicin on rat cardiac metabolism measured by hyperpolarized magnetic resonance spectroscopy. **a** Cartoon illustrating metabolites visible in the rat heart in vivo with magnetic resonance spectroscopy (MRS) after intravenous injection of hyperpolarized [1-^13^C]pyruvate (green) or [2-^13^C]pyruvate (blue). Representative spectra of a cardiac hyperpolarized [2-^13^C]pyruvate (**b**) or [1-^13^C]pyruvate (**c**) scan. Spectra are the sum of 30 s of data. **d** Cardiac bicarbonate:pyruvate ratio and **e**, lactate:pyruvate ratio of the sum of the first 30 s of MR spectra after intravenous injection of hyperpolarized [1-^13^C]pyruvate in male Wistar rats treated for six consecutive weeks with intravenous weekly injections of 4 mL kg^−1^ sterile saline or 2 mg kg^−1^ doxorubicin (DOX low) or treated for five consecutive weeks with intravenous weekly injections of 3 mg kg^−1^ DOX (DOX high). **f** Cardiac glutamate:pyruvate ratio and **g** acetyl-carnitine:pyruvate ratio of the sum of the first 30 s of MR spectra after intravenous injection of hyperpolarized [2-^13^C]pyruvate. **h** Orthogonal partial least squares discriminate analysis (OPLS-DA) of metabolomic data discriminating between the control group and the combined group of DOX high and DOX low (parameters for the OPLS-DA model: *R*^2^(*X*) = 76%, *R*^2^(*Y*) = 99%, *Q*^2^ = 59%, passed the random permutation test). **i** s-plot of OPLS-DA plot in **h** displaying metabolites according to their loadings and correlation with class membership. PCr phosphocreatine. Where applicable, box and whisker plots ranging from min to max value with the median indicated by horizontal line. Statistical comparison by two-way ANOVA with Tukey’s HSD correction method for multiple comparisons. *P < 0.05, **P < 0.01, ****P < 0.0001 compared to saline control group. ^$^Statistically significant difference between DOX high and DOX low group. Source data are provided in Supplementary Data 2.

At the time of CINE MRI, rats in this study also received two injections of hyperpolarized [1-^13^C]- and [2-^13^C]pyruvate, respectively, to assess myocardial carbohydrate metabolism and tricarboxylic acid (TCA) cycle flux in vivo (Fig. 2)^23,24^. In the high dose DOX group, there was a decrease in the bicarbonate:pyruvate ratio due to decreased pyruvate dehydrogenase (PDH)^28^ flux indicative of reduced carbohydrate oxidation, evident from week 3 onward (Fig. 2d). Anaerobic carbohydrate metabolism apparent through the lactate:pyruvate ratio was unchanged (Fig. 2e). In parallel with changes to PDH flux there was a marked decrease of tricarboxylic acid (TCA)-cycle derived glutamate at week 6 in both DOX groups, (Fig. 2f). Furthermore, DOX treatment led to a decrease in acetyl-carnitine labeling indicative of reduced acetyl-CoA buffering capacity^29^ in both DOX groups at week 6 (Fig. 2g). Overall, these changes in real-time in vivo metabolic fluxes demonstrate a decrease in oxidative mitochondrial carbohydrate metabolism in the DOX-treated hearts, which in the high dose DOX group preceded the onset of HF in week 6 measured by CINE MRI.

Plasma glucose levels did not change in either DOX group at any time point (Fig. S1c), suggesting that substrate supply was not responsible for this decrease in oxidative metabolism. At the 6 week timepoint, plasma lactate, nonesterified fatty acids (NEFA), and β-hydroxybutyrate were increased in the DOX high group (Fig. S1d–f). Carbohydrate oxidation in the heart is reciprocally controlled by fatty acid oxidation via the Randle cycle^30^, and decreased PDH flux at the 6 week timepoint may be influenced by increased fatty acid supply to the heart. Furthermore, while there was no decrease in PDH flux in the low dose DOX group at any time point in our study, these rats showed a decrease in apparent TCA cycle flux (glutamate:pyruvate ratio) after six injections at the final cumulative dose of 12 mg kg^−1^. This dose was reached in the high dose DOX group already after four injections, where a decrease in PDH flux was apparent. This suggests that higher individual DOX doses lead to accelerated cardiac damage resulting in a greater reduction in cardiac oxidative capacity than administration of lower doses over a prolonged time period. Interestingly, patients with metabolic syndrome are more likely to develop cardiac dysfunction after DOX chemotherapy, highlighting that whole-body and not just aberrant cardiac metabolism may play a critical role in the pathology of DOX-induced heart failure^31^.

After the last MRI scan rats were sacrificed, hearts were excised and metabolites extracted for analysis by LC-MS/MS. Orthogonal projections to latent structures (OPLS) analysis of the metabolomic dataset could clearly delineate the saline treated control group from the DOX groups (Figs. 2h and S2a). A loadings plot of the 90 aqueous metabolites and 25 acyl-carnitine species (Supplementary Data 1) revealed that a decrease in the cardiac pool sizes of the TCA cycle intermediate malate and TCA cycle-related glutamate as well as total carnitine, acetyl-carnitine and the adenine nucleotides NAD, AMP, ADP, and ATP and the PCr:ATP ratio in hearts from DOX-treated rats was driving this group distinction (Figs. 2i and S2b). A progressive decrease in the cardiac total adenine nucleotide pool was previously shown in a dog model of pacing-induced heart failure^32^. Therefore, these data further support the hypothesis that mitochondrial oxidative metabolism and energy generation is decreased in the rat heart following repeated DOX-treatment and that this drives the onset of heart failure in these rats.

### Mitochondrial number and metabolism are impaired in the DOX-treated rat heart

**Fig. 3:**
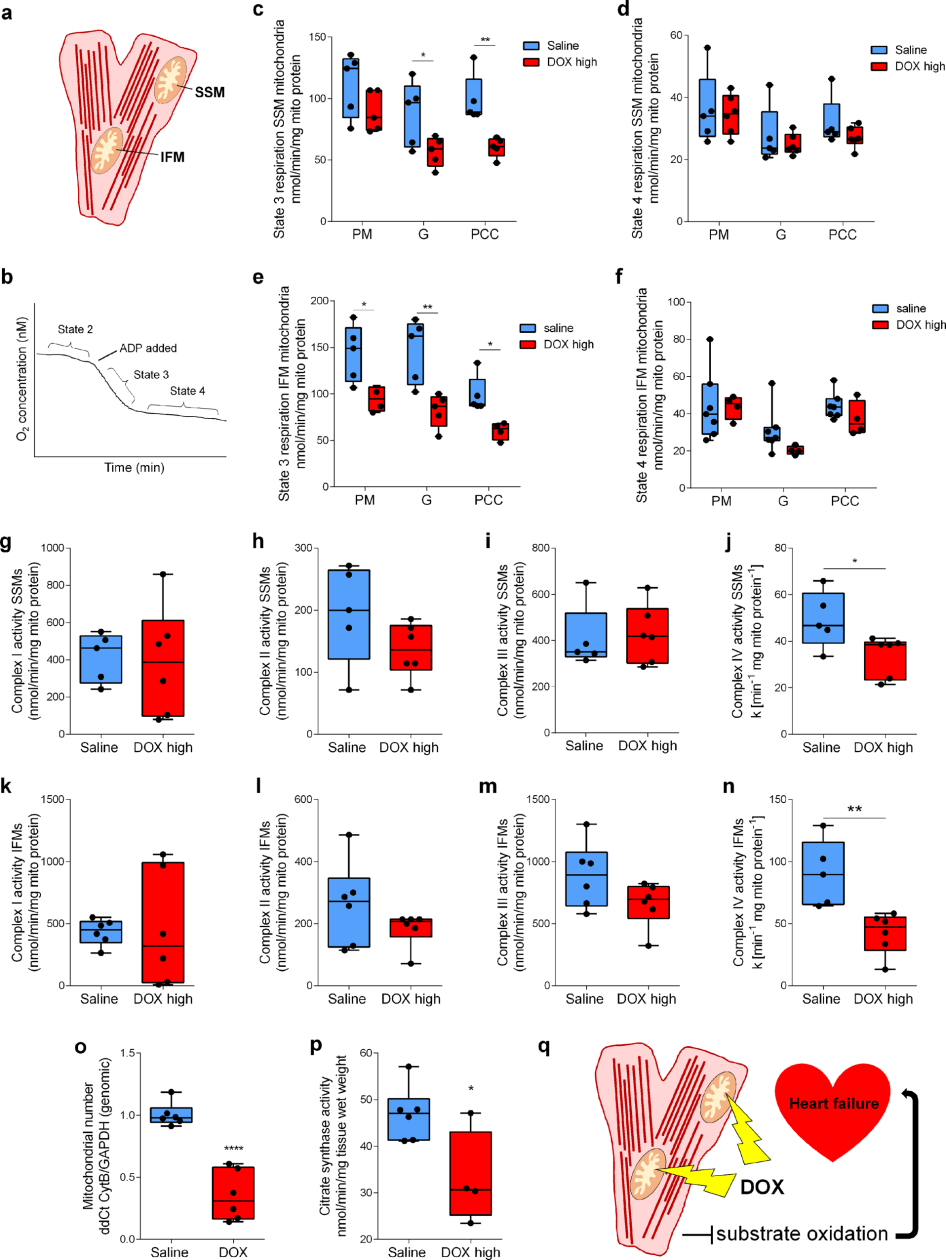
Cardiac mitochondrial function and number in doxorubicin-treated rats. **a** Cartoon depicting intracellular localization of sub-sarcolemmal (SSM) and interfibrillar (IFM) mitochondria inside cardiomyocytes. **b** Schematic showing mitochondrial oxygen consumption measurements where state 2 respiration is the background respiration in isolated mitochondria with substrate alone, state 3 depicts ADP-stimulated respiration and state 4 ADP-limited respiration after all ADP has been phosphorylated to ATP. **c**–**f** Oxygen consumption measurements (Clarke-style electrode) performed in isolated SSMs and IFMs (week 6) from male Wistar rats treated for 6 consecutive weeks with intravenous weekly injections of 4 mL kg^−1^ sterile saline or treated for 5 consecutive weeks with intravenous weekly injections of 3 mg kg^−1^ DOX (DOX high). State 3 and state 4 respiration with pyruvate + malate (PM), glutamate (G) or palmitoyl-CoA + carnitine (PCC) as a fuel in SSMs (**c**, **d**) and IFMs (**e**, **f**). Complex I–IV activity in the same SSMs (**g**–**j**) and IFMs (**k**–**n**) measured with spectrophotometric assays. Mitochondrial number assessed by **o**, qPCR analysis of a mitochondrial gene (cytochrome B; cytB) compared to a nuclear gene (glyceraldehyde-3-phosphate dehydrogenase; GAPDH) in genomic DNA-extracts from heart tissue and by **p**, citrate synthase activity in protein extracts from heart tissue. **q** Proposed mechanism that DOX treatment leads to mitochondrial loss and concomitant inadequate substrate oxidation leading to heart failure. Box and whisker plots ranging from min to max value with the median indicated by horizontal line. Some graphs do not start at *y* = 0 to allow for better visualization of the data spread. Statistical comparison by two-way ANOVA (**c**–**f**) with Tukey’s HSD correction method for multiple comparisons or by paired *t*-tests (**g**–**p**). *P < 0.05, **P < 0.01 compared to saline control group. Source data are provided in Supplementary Data 2.

A separate cohort of rats (cohort 2) was treated with either saline or high dose DOX as described above, with the rats sacrificed at week 6 and hearts excised for isolation of sub-sarcolemmal (SSM) and interfibrillar (IFM) mitochondria. Both SSM and IFM from high dose DOX hearts showed a decreased oxygen consumption rate with glutamate or palmitoyl-CoA + carnitine as substrates in state 3 but not state 4 respiration and IFM additionally showed a significant decrease with pyruvate + malate in state 3 (Fig. 3c–f). Electron transport chain (ETC) complex activity assays in SSM and IFM revealed no difference in complex I–III activity but there was a significant decrease in complex IV activity in both mitochondrial populations (Fig. 3g–n). In addition, there was a significant decrease in mitochondrial number in the high dose DOX group (Fig. 3o, p). Overall this suggests that DOX-HF is driven by a loss of mitochondria and a decrease in mitochondrial function driven by a decrease in complex IV activity (Fig. 3q).

### Oxidative stress is not a driving factor for cardiac dysfunction in this model of DOX-HF

Mitochondrial oxidative stress is thought to be a key factor for DOX-cardiotoxicity and reactive oxygen species can inhibit the ETC and damage mitochondria^33^. We therefore next wanted to established whether the mitochondrially-targeted antioxidant, MitoQ^34^, can prevent DOX-HF. It has previously been shown in animal models that MitoQ can protect the heart from ischemia/reperfusion injury^35^, and prevent loss of mitochondrial function associated with oxidative stress in a pressure overload model of heart failure^36^. MitoQ has also shown a protective effect on heart damage due to acute high doses of doxorubicin^37^ and interestingly, this damage was associated with reduced complex IV activity, which was restored by MitoQ treatment. To test whether MitoQ was also cardioprotective in this clinically relevant chronic model of DOX-HF, a separate high dose DOX group received MitoQ in their drinking water (0.5 mM) ad libitum 48 h before the first dose of DOX and then continuously until the end of the study. These concentrations of MitoQ have previously been shown to lead to steady-state levels of MitoQ in the rat heart that protects from hypertension-induced hypertrophy^38,39^. Here, MitoQ reached a tissue concentration of 3.7 ± 0.5 pmol g^−1^ in the rat heart after 6 weeks and MitoQ has been shown to protect cells from oxidative damage previously at these levels^40^. Therefore, the effect of MitoQ would indicate whether or not oxidative stress contributed to DOX-HF in this model. Surprisingly, we found no protective effect of MitoQ on either cardiac function (Fig. S3a), cardiac metabolic fluxes (S3b, c) or cardiac mitochondrial number (Fig. S3d). While we administered MitoQ ad libitum in drinking water, MitoQ in other studies has been delivered twice weekly i.p.^37^. This may lead to different concentrations of MitoQ in the heart and therefore different protective capacity. Furthermore, we could not find any evidence of either long-term oxidative stress (Fig. S4a–c) or acute reactive oxygen species production (Fig. S4d) in our DOX-treated rat hearts. Likewise, we conducted RNAseq of RNA extracts from DOX high and saline-treated control hearts, which showed no evidence that gene sets associated with either oxidative stress or reactive oxygen species were altered in our model (Table S1). However, we only assessed hydrogen peroxide production directly with MitoB but did not assess short-lived ROS such as superoxide or peroxynitrite in our model. Nevertheless, overall our data indicate that oxidative stress in this chronic and clinically relevant model of DOX-HF is not driving the pathology.

### Conclusion

In summary, we have shown here that hyperpolarized MRS can serve as a unique marker of mitochondrial integrity and oxidative mitochondrial carbohydrate metabolism and thereby detect DOX-induced cardiotoxicity before functional decline. We furthermore show that oxidative stress in this model of DOX-HF is not responsible for the loss of mitochondria and the onset of HF. The first images of real-time metabolic flux in the human heart have been published using hyperpolarized MRI^21^ and exciting clinical research is ongoing to understand the role of cardiac metabolism in cardiovascular disease^20^, opening up the potential to apply this technology to the study of both DOX-HF and HF of other aetiologies in patients.

## Methods

### Animal studies

All animal experiments conformed to Home Office Guidance on the Operation of the Animals (Scientific Procedures) Act, 1986 and were approved by a local ethics committee. Most animal models of DOX-cardiotoxicity employ high doses of DOX, far above the equivalent recommended life-time dose for patients^41^. Furthermore these are often administered intraperitoneally in a single dose rather than infused i.v. in repeat doses as is done clinically. Intraperitoneal injections of DOX can lead to severe, painful inflammation and fibrosis^42^, which, as well as being deleterious from an animal welfare point of view, may lead to alterations in food intake and hence metabolic status as well as confounding DOX-specific effects due to inflammation. We therefore chose to deliver DOX intravenously through a tail vein with small individual doses administered over several weeks to best mimic patient treatment schemes. Three separate cohorts of age and weight-matched male Wistar rats (6–8 weeks, 238 ± 36 g (standard deviation)) were used. For cohort 1, rats were split into four groups and treated weekly for 6 weeks with i.v. injection of either 4 mL kg^−1^ sterile saline (*n* = 20) or 2 mg kg^−1^ DOX (Apollo Scientific) dissolved in sterile saline (Dox low, *n* = 12) or for five weeks with 3 mg kg^−1^ DOX (Dox high, *n* = 8) or 3 mg kg^−1^ DOX + 0.5 mM MitoQ^34^ in drinking water ad libitum for the duration of the study (DOX high + MitoQ, *n* = 6). Rats were weighed weekly during the study. At weeks 1, 3, and 6 rats were anaesthetized with 2% isoflurane in medical oxygen and blood samples were taken for analysis of plasma cardiac troponin I (cTNI) with a commercially available ELISA kit (CTNI-2-HSP, Life Diagnostics, West Chester, PA, USA) and plasma lactate dehydrogenase (LDH) with a commercially available kit (ABX Pentra, Horiba, Irvine California, CA, USA). Glucose, lactate, nonesterified fatty acids (NEFA), and β-hydroxybutyrate were measured in the plasma with a commercially available kits (Randox, Crumlin, UK). Rats then underwent an MR imaging protocol (see below). At the end of the study, rats were sacrificed and their epididymal fat pads weighed and tibia length measured. A separate cohort (cohort 2) was treated as above with either saline or 3 mg kg^−1^ DOX (*n* = 6 for both groups) and used for mitochondrial isolation and functional analysis. Cohort 3 was treated as above with either saline or 3 mg kg^−1^ DOX once (*n* = 4 each) or weekly for 5 consecutive weeks (*n* = 4 each) and used for measurements of intramitochondrial hydrogen peroxide production (see “Measurements of oxidative stress” section).

### Cardiac functional and metabolic analysis with MRI, MRS, and LC-MS/MS

After blood sampling, and during the same anesthesia, rats from cohort 1 were used for functional CINE MR imaging (MRI) and real-time metabolic flux measurements with hyperpolarized [1-^13^C]– and [2-^13^C]pyruvate MRS, performed on a 7 T preclinical MRI system (Varian) as previously described^43^. The order of CINE MRI and hyperpolarized [1-^13^C]– and [2-^13^C]pyruvate MRS scans was randomized between different rats. For the hyperpolarized experiments, 1 mL of 80 mM [1-^13^C]– or [2-^13^C]pyruvate was injected into the tail vain over 10 s. ^13^C MR spectra were acquired from the heart every second for 60 s using a 72 mm dual-tuned birdcage volume transmit ^1^H/^13^C coil and a ^13^C two-channel surface receive coil (Rapid Biomedical; 15° hard pulse; 13.2 kHz bandwidth for [1-^13^C]pyruvate and 17.6 kHz for [2-^13^C]pyruvate). Multicoil spectra were manually added in phase, and the first 30 s of spectra from the first appearance of the pyruvate peak were summed and quantified with AMARES/jMRUI as described previously^44^. After the last MRI scan, rats from cohort 1 were sacrificed and hearts excised and rapidly snap-frozen by freeze clamping with liquid nitrogen-cooled Wallenberger tongs. Metabolites were extracted from 50 mg samples of the hearts from cohort 1 (excluding DOX high + MitoQ group) with 2:1 chloroform:methanol using metal-bead containing tubes and a Precellys tissue homogenizer (Bertin Instruments, Montigny-le-Bretonneux, France) and metabolomic analysis was performed with liquid chromatography tandem mass spectrometry (LC-MS/MS)^45^. Hearts from rats treated with MitoQ were extracted with 60% acetonitrile containing 0.1% formic acid using metal-bead containing tubes and a Precellys tissue homogenizer, and MitoQ levels were assessed by LC-MS/MS^46^.

### Mitochondrial analysis

Rats from cohort 2 were sacrificed under terminal isoflurane anesthesia (5% in medical oxygen) at week 6 and their hearts excised. A ~100 mg piece of the heart apex was freeze clamped and from the remaining tissue subsarcolemmal (SSM) and interfibrillar (IFM) mitochondria were isolated^47^. Oxygen consumption rates were assessed in mitochondria (0.15 mg mitochondrial protein per experiment) at 30 °C with a Clarke-type oxygen electrode (Strathkelvin Instruments Ltd, Glasgow, UK) using pyruvate + malate, glutamate or palmitoyl-CoA + carnitine as substrates. Mitochondrial complex I–IV activities were assessed in the same isolated mitochondria spectrophotometrically as previously described^48^. From the frozen apex, 30 mg were used for DNA extraction using a DNeasy^®^ blood and tissue extraction kit (Qiagen, Venlo, Netherlands). Quantitative real-time PCR was performed using the Power SYBR Green PCR Master Mix and a Step-one Plus Real-Time PCR system (Thermo Fisher Scientific) to assess copy numbers for the mitochondrial gene cytochrome b (cytB) and the nuclear-encoded gene glyceraldehyde-3-phosphate dehydrogenase (GAPDH). Primer sequences (5′–3′) were as follows: GAPDH sense, AGTATGTCGTG GAGTCTACTGGTG; GAPDH anti-sense, TGAGTTGTCATATTTCTCGTGGTT; cytB sense, GGGTATGTACTCCCATGAGGAC; cytB anti-sense, CCTCCTCAGATTCATTCGAC. The data were analyzed with the comparative *C*_t_ method^49^ with relative cytB copy number as a marker of mitochondrial number. The remaining heart apex tissue was ground to a fine powder under liquid nitrogen and used for protein extraction, and citrate synthase activity measurements using spectrophotometric analysis as described previously^48^.

### Measurements of oxidative stress

Oxidative stress in the heart was assessed in three separate ways: Heart tissue from cohort 1 were analyzed for long-term oxidative stress with commercially available ELISA kits for nitrotyrosine residues as a marker of protein nitrosylation and for 8-hydroxy-2′-deoxyguanosine as a marker of DNA oxidation (ab113848 and ab201734, abcam, Cambridge, UK). In vivo intramitochondrial hydrogen peroxide levels were measured in cohort 3 with the mitochondrially targeted hydrogen peroxide-sensor, MitoB^50^. Rats were anaesthetized with 2% isofluorane in medical oxygen and injected intravenously with 1 nmol g^−1^ body weight MitoB in sterile saline either immediately after i.v. injection of saline or 3 mg kg^−1^ DOX (acute stress) or 48 h after injection of saline or 3 mg kg^−1^ DOX in week 1, 3, and 5 (chronic stress). Rats were re-anaesthetized 4 h after MitoB injection with 5% isoflurane in medical oxygen and hearts excised and rapidly freeze-clamped with liquid nitrogen-cooled Wallenberger tongs. Around 50 mg of heart tissue were extracted with ice-cold 60% acetonitrile containing 0.1% formic acid using metal-bead containing tubes and a Precellys tissue homogenizer (Bertin Instruments, Montigny-le-Bretonneux, France). Extracts were analyzed as previously described by LC-MS/MS^50^, with the ratio of the hydrogen peroxide-dependent oxidized product MitoP and MitoB as a marker of mitochondrial hydrogen peroxide levels.

### RNA extraction and RNAseq library preparation

RNA was extracted with RNeasy® Fibrous Tissue Mini kit (Qiagen, Manchester, UK) using ~30 mg of snap-frozen heart tissue from the saline and DOX high group of cohort 1. Material was quantified using RiboGreen (Invitrogen) on the FLUOstar OPTIMA plate reader (BMG Labtech) and the size profile and integrity analysed on the 2200 (Agilent, RNA ScreenTape). RIN estimates for all samples were above 7. Input material was normalized to equal input of 100 ng prior to library preparation. Polyadenylated transcript enrichment and strand specific library preparation was completed using NEBNext Ultra II mRNA kit (NEB) following manufacturer’s instructions. Libraries were amplified on a Tetrad (Bio-Rad) using in-house unique dual indexing primers (based on 10.1186/1472-6750-13-104). Individual libraries were normalised using Qubit, and the size profile was analysed on the 2200 or 4200 TapeStation. Individual libraries were normalised and pooled together accordingly. The pooled library was diluted to ~10 nM for storage. The 10 nM library was denatured and further diluted prior to loading on the sequencer. Paired end sequencing was performed using a HiSeq4000 75 bp platform (Illumina, HiSeq 3000/4000 PE Cluster Kit and 150 cycle SBS Kit), generating a raw read count of 22 million read pairs per sample.

### RNAseq mapping and counts

RNAseq read pairs were aligned to Rattus norvegicus reference genome, Rnor_6.0 using a splice-aware aligner, Hisat2 version-2.0.4^51^. Gene annotation files were downloaded in GTF format from Ensembl, release 81^52^. Read fragments mapping to annotated exon features were quantified with featureCounts^53^, part of subread-v1.5.0^54^, using default parameters. Values for duplication rates and median 3′ bias were estimated using MarkDuplicates, and CollectRnaSeqMetrics respectively, both implemented in Picard tools v1.92^55^. Normalized read counts and count based metrics were obtained using in-house R scripts^56^, R core tools v3.1.0. Count tables were then analysed with freely available gene set enrichment analysis (GSEA) software (Broad Institute, Inc., Massachusetts Institute of Technology, and Regents of the University of California) using the Molecular Signatures Database v7.0 (gene set C5 Biological Processes).

### Statistics and reproducibility

Statistical analysis was performed in Prism 6.0 (GraphPad, La Jolla, CA, US). Unpaired Student’s *t*-tests, one-way or two-way ANOVA with Tukey’s HSD adjustment method for multiple comparisons was used and performed as indicated in the figure legends. Significance was assumed at *P* < 0.05. Only significances from multiple comparisons are displayed in the figures and not the ANOVA interactions. Multivariate statistics for metabolomic analysis was performed within Simca version 15 (Umetrics, Umea, Sweden). Initially principal components was performed to identify samples that were outliers. To maximize separation associated with class membership (e.g., control group versus drug-treated groups), orthogonal partial least squares discriminant analysis (OPLS-DA) was performed. Model validity was assessed by random permutation tests and the metabolites most important for class discrimination identified using the S-plot showing the OPLS-DA loadings against the correlation coefficients for class membership. Significance in gene set enrichment between DOX high and saline control groups were assumed at a false discovery rate (FDR) *q*-value < 0.25 with a more stringent cut off at a family-wise error rate (FWER) p-value < 0.05. All experiments were either in vivo or derived from animal tissue and individual data points are shown in graphs. Multiple animals were used per group as indicated in the “Methods” section but ex vivo experiments were only performed once with an appropriate *n*-number and not replicated in separate identical experiments. Figure legends display details on statistical tests.

### Reporting summary

Further information on research design is available in the Nature Research Reporting Summary linked to this article.

## Supplementary information

Supplementary Information

Description of Additional Supplementary Files

Reporting Summary

## Data Availability

The source data underlying Figs. 1b–e, g–m, 2d–g, 3c–p, S1a–f, S3a–d, and S4a–d are provided in Supplementary Data 2, which furthermore contains the full GSEA data set underlying Table S1 (GSEA C5 Biological Processes). The full RNAseq dataset can be accessed from Gene Expression Omnibus using the accession code GSE154603. All other data are available from authors upon reasonable request.

## References

[1] Lipshultz SE (2006). Exposure to anthracyclines during childhood causes cardiac injury. Semin. Oncol..

[2] Cardinale D (2015). Early detection of anthracycline cardiotoxicity and improvement with heart failure therapy. Circulation.

[3] Hahn, V. S., Lenihan, D. J. & Ky, B. Cancer therapy-induced cardiotoxicity: Basic mechanisms and potential cardioprotective therapies. *J. Am. Heart Assoc*. **3**, e000665 (2014). 10.1161/JAHA.113.000665 PMC4187516 24755151

[4] Kalyanaraman B (2002). Doxorubicin-induced apoptosis: Implications in cardiotoxicity. Mol. Cell. Biochem..

[5] Li DL, Hill JA (2014). Cardiomyocyte autophagy and cancer chemotherapy. J. Mol. Cell. Cardiol..

[6] Koleini N, Kardami E (2017). Autophagy and mitophagy in the context of doxorubicin-induced cardiotoxicity. Oncotarget.

[7] Varga ZV, Ferdinandy P, Liaudet L, Pacher P (2015). Drug-induced mitochondrial dysfunction and cardiotoxicity. Am. J. Physiol..

[8] Šimůnek T (2009). Anthracycline-induced cardiotoxicity: overview of studies examining the roles of oxidative stress and free cellular iron. Pharmacol. Rep..

[9] Swain SM, Vici P (2004). The current and future role of dexrazoxane as a cardioprotectant in anthracycline treatment: expert panel review. J. Cancer Res. Clin. Oncol..

[10] Yi LL (2007). Topoisomerase IIβ-mediated DNA double-strand breaks: Implications in doxorubicin cardiotoxicity and prevention by dexrazoxane. Cancer Res..

[11] Lipshultz SE (2014). Dexrazoxane for reducing anthracycline-related cardiotoxicity in children with cancer: an update of the evidence. Prog. Pediatr. Cardiol..

[12] Zhang S (2012). Identification of the molecular basis of doxorubicin-induced cardiotoxicity. Nat. Med..

[13] Dhingra R (2014). Bnip3 mediates doxorubicin-induced cardiac myocyte necrosis and mortality through changes in mitochondrial signaling. Proc. Natl Acad. Sci. USA.

[14] Mukhopadhyay, P. et al. Role of superoxide, nitric oxide, and peroxynitrite in doxorubicin-induced cell death in vivo and in vitro. *Am. J. Physiol.* **296**, H1466–H1483 (2009). 10.1152/ajpheart.00795.2008 PMC2685360 19286953

[15] Tokarska-Schlattner M, Zaugg M, Zuppinger C, Wallimann T, Schlattner U (2006). New insights into doxorubicin-induced cardiotoxicity: the critical role of cellular energetics. J. Mol. Cell. Cardiol..

[16] Neubauer S (2007). The failing heart—an engine out of fuel. N. Engl. J. Med..

[17] Piramoon S, Aberoomand Azar P, Saber Tehrani M, Mohammadiazar S, Tavassoli A (2017). Solid-phase nanoextraction of polychlorinated biphenyls in water and their determination by gas chromatography with electron capture detector. J. Sep. Sci..

[18] Ardenkjaer-Larsen JH (2003). Increase in signal-to-noise ratio of >10,000 times in liquid-state NMR. Proc. Natl Acad. Sci. USA.

[19] Timm KN, Miller JJ, Henry JA, Tyler DJ (2018). Cardiac applications of hyperpolarised magnetic resonance. Prog. Nucl. Magn. Reson. Spectrosc..

[20] Rider OJ (2020). Noninvasive in vivo assessment of cardiac metabolism in the healthy and diabetic human heart using hyperpolarized 13C MRI. Circ. Res..

[21] Cunningham CH (2016). Hyperpolarized 13C metabolic MRI of the human heart: initial experience. Circ. Res..

[22] Lewis AJM (2018). Noninvasive immunometabolic cardiac inflammation imaging using hyperpolarized magnetic resonance. Circ. Res..

[23] Schroeder MA (2008). In vivo assessment of pyruvate dehydrogenase flux in the heart using hyperpolarized carbon-13 magnetic resonance. Proc. Natl Acad. Sci. USA.

[24] Schroeder MA (2009). Real-time assessment of Krebs cycle metabolism using hyperpolarized 13C magnetic resonance spectroscopy. FASEB J..

[25] Zhang Y (2014). Doxorubicin induces sarcoplasmic reticulum calcium regulation dysfunction via the decrease of SERCA2 and phospholamban expressions in rats. Cell Biochem. Biophys..

[26] Moulin M (2015). Sexual dimorphism of doxorubicin-mediated cardiotoxicity potential role of energy metabolism remodeling. Circulation.

[27] Tassan-Mangina S (2006). Tissue Doppler imaging and conventional echocardiography after anthracycline treatment in adults: early and late alterations of left ventricular function during a prospective study. Eur. J. Echocardiogr..

[28] Atherton HJ (2011). Validation of the in vivo assessment of pyruvate dehydrogenase activity using hyperpolarised 13C MRS. NMR Biomed..

[29] Schroeder MA (2012). The cycling of acetyl-coenzyme A through acetylcarnitine buffers cardiac substrate supply: A hyperpolarized 13C magnetic resonance study. Circ. Cardiovasc. Imaging.

[30] Hue, L. & Taegtmeyer, H. The randle cycle revisited: a new head for an old hat. *Am. J. Physiol. Endocrinol. Metab*. **297** E578–E591 (2009). 10.1152/ajpendo.00093.2009 PMC2739696 19531645

[31] Armstrong GT (2015). Comprehensive echocardiographic detection of treatment-related cardiac dysfunction in adult survivors of childhood cancer: results from the St. Jude Lifetime Cohort Study. J. Am. Coll. Cardiol..

[32] Shen W (1999). Progressive loss of myocardial ATP due to a loss of total purines during the development of heart failure in dogs: a compensatory role for the parallel loss of creatine. Circulation.

[33] Kirkinezos IG, Moraes CT (2001). Reactive oxygen species and mitochondrial diseases. Semin. Cell Dev. Biol..

[34] Kelso GF (2001). Selective targeting of a redox-active ubiquinone to mitochondria within cells: Antioxidant and antiapoptotic properties. J. Biol. Chem..

[35] Adlam VJ (2005). Targeting an antioxidant to mitochondria decreases cardiac ischemia-reperfusion injury. FASEB J..

[36] Ribeiro Junior RF (2018). MitoQ improves mitochondrial dysfunction in heart failure induced by pressure overload. Free Radic. Biol. Med..

[37] Chandran K (2009). Doxorubicin inactivates myocardial cytochrome c oxidase in rats: Cardioprotection by Mito-Q. Biophys. J..

[38] Graham D (2009). Mitochondria-targeted antioxidant mitoq10 improves endothelial function and attenuates cardiac hypertrophy. Hypertension.

[39] Smith RAJ, Porteous CM, Gane AM, Murphy MP (2003). Delivery of bioactive molecules to mitochondria in vivo. Proc. Natl Acad. Sci. USA.

[40] Jauslin ML (2003). Mitochondria-targeted antioxidants protect Friedreich Ataxia fibroblasts from endogenous oxidative stress more effectively than untargeted antioxidants. FASEB J..

[41] Moslehi JJ (2016). Cardiovascular toxic effects of targeted cancer therapies. N. Engl. J. Med..

[42] Goodman MD, McPartland S, Detelich D, Saif MW (2016). Chemotherapy for intraperitoneal use: a review of hyperthermic intraperitoneal chemotherapy and early post-operative intraperitoneal chemotherapy. J. Gastrointest. Oncol..

[43] Dodd MS (2012). In vivo alterations in cardiac metabolism and function in the spontaneously hypertensive rat heart. Cardiovasc. Res..

[44] Vanhamme L, Van Den Boogaart A, Van Huffel S (1997). Improved method for accurate and efficient quantification of MRS data with use of prior knowledge. J. Magn. Reson..

[45] Wang X, West JA, Murray AJ, Griffin JL (2015). Comprehensive metabolic profiling of age-related mitochondrial dysfunction in the high-fat-fed ob/ob mouse heart. J. Proteome Res..

[46] Li Y, Zhang H, Fawcett JP, Tucker IG (2007). Quantitation and metabolism of mitoquinone, a mitochondria-targeted antioxidant, in rat by liquid chromatography/tandem mass spectrometry. Rapid Commun. Mass Spectrom..

[47] Palmer W (1977). Biochemical interfibrillar muscle*. Biol. Chem..

[48] Heather LC (2012). Metabolic adaptation to chronic hypoxia in cardiac mitochondria. Basic Res. Cardiol..

[49] Schmittgen TD, Livak KJ (2008). Analyzing real-time PCR data by the comparative CT method. Nat. Protoc..

[50] Cochemé HM (2011). Measurement of H2O2 within living drosophila during aging using a ratiometric mass spectrometry probe targeted to the mitochondrial matrix. Cell Metab..

[51] Kim D, Langmead B, Salzberg SL (2015). HISAT: a fast spliced aligner with low memory requirements. Nat. Methods.

[52] Cunningham F (2015). Ensembl 2015. Nucleic Acids Res..

[53] Liao Y, Smyth GK, Shi W (2014). FeatureCounts: an efficient general purpose program for assigning sequence reads to genomic features. Bioinformatics.

[54] Liao Y, Smyth GK, Shi W (2013). The Subread aligner: fast, accurate and scalable read mapping by seed-and-vote. Nucleic Acids Res..

[55] Picard-tools. https://broadinstitute.github.io/picard/. Accessed 26 March 2020).

[56] R Core Team. *R: A Language and Environment for Statistical Computing* (R Core Team, Vienna, 2014).

