# Supplementary material for: Early detection of doxorubicin-induced cardiotoxicity in rats by its cardiac metabolic signature assessed with hyperpolarized MRI
Source: Commun Biol. 2020 Nov 19;3:692. doi: 10.1038/s42003-020-01440-z (PMC7678845; doi:10.1038/s42003-020-01440-z)
Supplement: Supplementary file 1 — Supplementary Information [file 42003_2020_1440_MOESM1_ESM.pdf]

Supplementary Information

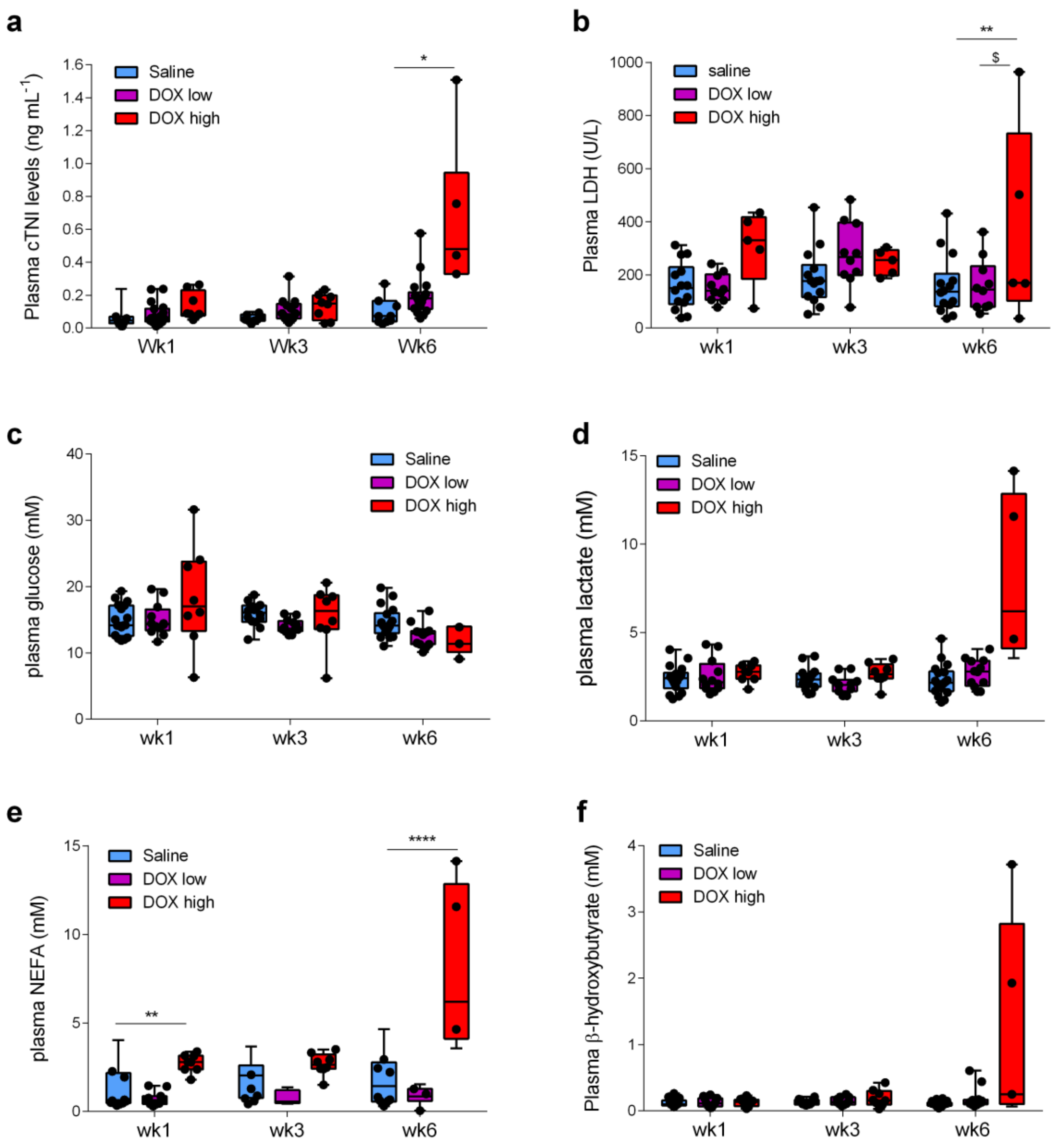

Figure S1 DOX increases plasma markers of cardiac damage and perturbs plasma substrate levels. Plasma **a**, cardiac troponin I (cTNI) **b**, lactate dehydrogenase (LDH) **c,** glucose **d,** lactate **e,** non-esterified fatty acids (NEFA) and **f,** β-hydroxybutyrate in DOX-treated rats at weeks 1, 3 and 6 of the study. Box and whisker plots ranging from min to max value. Statistical comparison by two-way ANOVA with Tukey's HSD correction method for multiple comparisons. *P<0.05, **P<0.01, ****P<0.0001 compared to saline control group. [$]Statistically

significant difference between DOX high and DOX low group. Source data are provided in Supplementary Data 2.

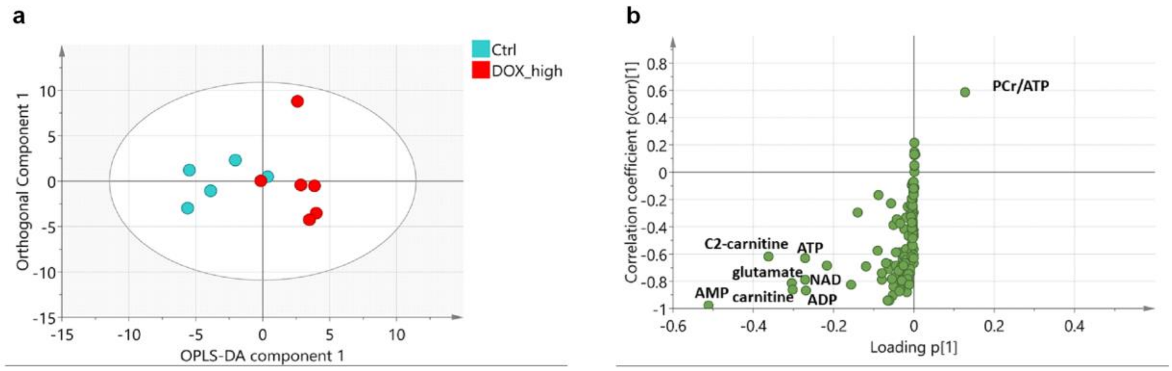

Figure S2 Metabolomic analysis of cardiac tissue confirms reduced TCA cycle intermediates and high energy phosphates indicative of mitochondrial dysfunction. a, Orthogonal partial least squares discriminate analysis (OPLS-DA) of the metabolomics data comparing the high dose DOX group with their respective controls (parameters for the OPLS-DA model: $R^2(X)=85\%$, $R^2(Y)=73\%$, $Q^2=48\%$; passed the random permutation test). **b,** s-plot of OPLS-DA displaying metabolites according to their loadings and correlation with class membership. PCr – phosphocreatine, C2-carnitine – acetyl-carnitine**.**

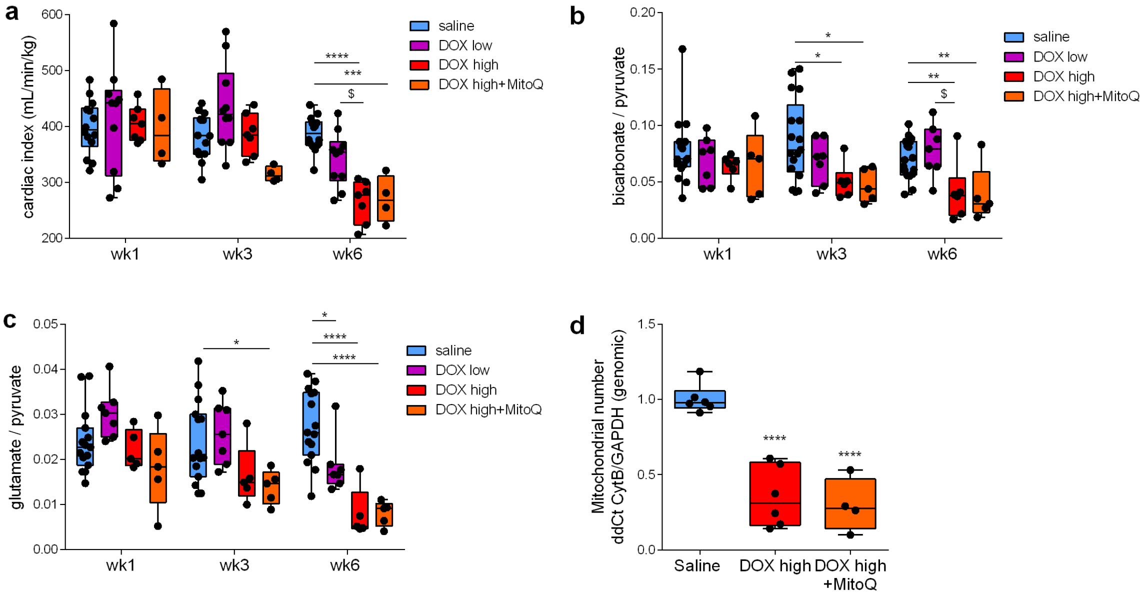

Figure S3 The mitochondrially-targeted antioxidant MitoQ cannot prevent DOX-HF. A separate cohort of male Wistar rats was treated for 5 consecutive weeks with i.v. injections of 3 mg kg^{-1} DOX (n=6) and retrieved 0.5 mM MitoQ in drinking water *ad libitum* throughout the 6 week study and from 48 h before the first DOX injection. Cardiac function measurements by CINE MRI and cardiac metabolic fluxes by hyperpolarized [1-^{13}C]pyruvate and [2-^{13}C]pyruvate MRS were performed at weeks 1, 3 and 6. Following MRI at week, hearts were excised and rapidly snap frozen for DNA extraction and mitochondrial number assessment by qPCR. **a**, cardiac index, **b**, cardiac bicarbonate:pyruvate **c**, glutamate:pyruvate ratio at all three time points. **d**, mitochondrial number assessed by comparative gene copy number of the mitochondrial gene, cytochrome b (cytB) compared to the nuclear gene GAPDH. Data were combined with data from rat cohort 1 represented in figures 1-3 (DOX high and saline control groups). Box and whisker plots ranging from min to max value with the mean indicated by horizontal line. Some graphs do not start at y=0 to allow for better visualization of the data spread. Statistical comparison by two-way ANOVA (**a-c**) or one-way ANOVA (**d)** with Tukey's HSD correction method for multiple comparisons. *P<0.05, **P<0.01, ***P<0.001, ****P<0.0001 compared to saline control group. $^{\$}$Statistically significant difference between DOX high and DOX low group. Source data are provided in Supplementary Data 2.

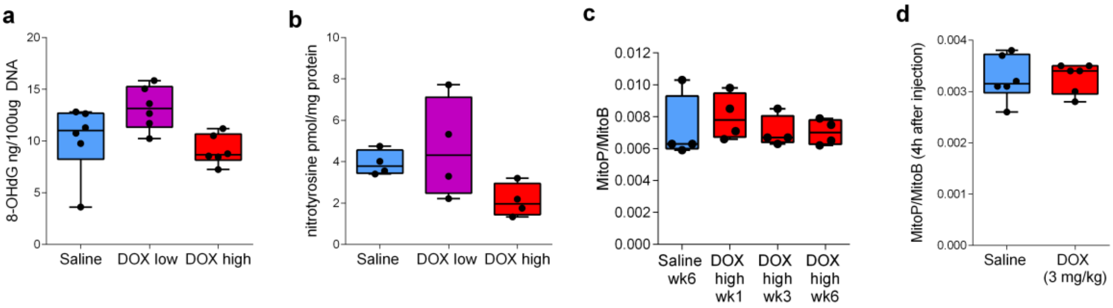

Figure S4 No evidence of oxidative stress in hearts from doxorubicin treated rats. a-c, Long-term cardiac oxidative stress in male Wistar rats treated for 6 consecutive weeks with intravenous weekly injections of 4 mL kg^{-1} sterile saline or 2 mg kg^{-1} doxorubicin (DOX low) or treated for 5 consecutive weeks with intravenous weekly injections of 3 mg kg^{-1} DOX (DOX high). **a**, DNA oxidation assessed by 8-hydroxy-2'-deoxyguanosine levels in heart DNA-extracts. **b**, protein nitrosylation measured by nitrotyrosine levels in heart protein-extracts. **c**, mitochondrial hydrogen peroxide levels assessed by the irreversible hydrogen peroxide mediated oxidation of the mitochondrially targeted probe, MitoB, to its oxidized form, MitoP, 4 hours after intravenous injection of MitoB, measured by liquid chromatography tandem mass spectrometry in heart tissue extracts. **d**, mitochondrial hydrogen peroxide levels in male Wistar rats measured as in **c** but 4 hours after injection of either 4 mL kg^{-1} sterile saline or 3 mg kg^{-1} DOX for acute assessment. Box and whisker plots ranging from min to max value with the median indicated by horizontal line. Source data are provided in Supplementary Data 2.

Table S1 RNAseq GSEA analysis DOX high vs saline (FDR< 0.25)

Molecular Signatures Database v7.0 gene set (C5 Biological Processes)	FDR q	FWER p
GO_CELLULAR_RESPONSE_TO_REACTIVE_OXYGEN_SPECIES	0.626	0.97
GO_REGULATION_OF_REACTIVE_OXYGEN_SPECIES_METABOLIC_PROCESS	0.629	0.978
GO_CELLULAR_RESPONSE_TO_OXYGEN_CONTAINING_COMPOUND	0.644	0.971
GO_RESPONSE_TO_OXYGEN_CONTAINING_COMPOUND	0.645	0.971
GO_POSITIVE_REGULATION_OF_REACTIVE_OXYGEN_SPECIES_METABOLIC_PROCESS	0.668	0.993
GO_RESPONSE_TO_OXYGEN_LEVELS	0.680	0.995
GO_CELLULAR_RESPONSE_TO_OXYGEN_LEVELS	0.689	0.999
GO_CELLULAR_RESPONSE_TO_OXIDATIVE_STRESS	0.696	0.999
GO_RESPONSE_TO_OXIDATIVE_STRESS	0.706	0.999
GO_REGULATION_OF_REACTIVE_OXYGEN_SPECIES_BIOSYNTHETIC_PROCESS	0.714	0.999
GO_RESPONSE_TO_REACTIVE_OXYGEN_SPECIES	0.723	0.999
GO_POSITIVE_REGULATION_OF_REACTIVE_OXYGEN_SPECIES_BIOSYNTHETIC_PROCESS	0.749	1
GO_REACTIVE_OXYGEN_SPECIES_METABOLIC_PROCESS	0.819	1
