# Supplementary material for: Early detection of doxorubicin-induced cardiotoxicity in rats by its cardiac metabolic signature assessed with hyperpolarized MRI
Source: Commun Biol. 2020 Nov 19;3:692. doi: 10.1038/s42003-020-01440-z (PMC7678845; doi:10.1038/s42003-020-01440-z)
Supplement: Supplementary file 2 — Description of Additional Supplementary Files [file 42003_2020_1440_MOESM2_ESM.pdf]

Description of Additional Supplementary Files

File Name: Supplementary Data 1
Description: Metabolomics of aqueous metabolites and acyl-carnitine species in heart tissue extracts

File Name: Supplementary Data 2
Description: Source Data underlying Figures 1b-e and g-m, 2d-g, 3c-p, S1a-f, S3a-d and S4a-d
