# Supplementary material for: Early detection of doxorubicin-induced cardiotoxicity in rats by its cardiac metabolic signature assessed with hyperpolarized MRI
Source: Commun Biol. 2020 Nov 19;3:692. doi: 10.1038/s42003-020-01440-z (PMC7678845; doi:10.1038/s42003-020-01440-z)
Supplement: Supplementary file 3 — Reporting Summary [file 42003_2020_1440_MOESM3_ESM.pdf]

nature research

Corresponding author(s): Kerstin N Timm

Last updated by author(s): Sep 23, 2020

Reporting Summary

Nature Research wishes to improve the reproducibility of the work that we publish. This form provides structure for consistency and transparency in reporting. For further information on Nature Research policies, see Authors & Referees and the Editorial Policy Checklist.

Statistics

For all statistical analyses, confirm that the following items are present in the figure legend, table legend, main text, or Methods section.

n/a	Confirmed	
☐	☒	The exact sample size (n) for each experimental group/condition, given as a discrete number and unit of measurement
☐	☒	A statement on whether measurements were taken from distinct samples or whether the same sample was measured repeatedly
☐	☒	The statistical test(s) used AND whether they are one- or two-sided<br>*Only common tests should be described solely by name; describe more complex techniques in the Methods section.*
☒	☐	A description of all covariates tested
☐	☒	A description of any assumptions or corrections, such as tests of normality and adjustment for multiple comparisons
☐	☒	A full description of the statistical parameters including central tendency (e.g. means) or other basic estimates (e.g. regression coefficient) AND variation (e.g. standard deviation) or associated estimates of uncertainty (e.g. confidence intervals)
☐	☒	For null hypothesis testing, the test statistic (e.g. F, t, r) with confidence intervals, effect sizes, degrees of freedom and P value noted<br>*Give P values as exact values whenever suitable.*
☒	☐	For Bayesian analysis, information on the choice of priors and Markov chain Monte Carlo settings
☒	☐	For hierarchical and complex designs, identification of the appropriate level for tests and full reporting of outcomes
☒	☐	Estimates of effect sizes (e.g. Cohen's d, Pearson's r), indicating how they were calculated

Our web collection on statistics for biologists contains articles on many of the points above.

Software and code

Policy information about availability of computer code

Data collection	For CINE MRI and hyperpolarized MRS acquisition VNMRJ v4.2 revision A was used. For ELISA and enzyme assay acquisition a FLUOstar Omega microplate reader spectrophotometer software v5.11 R3 was used. Plasma analysis of LDH and TAG was performed with Pentra c400 from Horiba. qPCR data was acquired with StepOnePlus v2.3.
Data analysis	For MRS analysis JMRUI 5.2 was used. CINE MRI analysis was performed in ImageJ. Oxygen consumption measurements were acquired and analyzed with a Clarke style electrode and associated software Strathkelvin 782 v4.1 . Spectrophotometric and qPCR data were analyzed with Microsoft Office Professional 2013 Excel. Mass spectrometry data were analyzed using Xcalibur v3.0. Statistical analysis was performed in GraphPad Prism. Multivariat statistical analysis of metabolomic data was performed in Simca v15. For RNAseq analysis normalized read counts and count based metrics were obtained using in-house R scripts, R core tools v3.1.0. Count tables were then analysed with freely available gene set enrichment analysis (GSEA) software (Broad Institute, Inc., Massachusetts Institute of Technology, and Regents of the University of California) using the Molecular Signatures Database v7.0 (gene set C5 Biological Processes)

For manuscripts utilizing custom algorithms or software that are central to the research but not yet described in published literature, software must be made available to editors/reviewers. We strongly encourage code deposition in a community repository (e.g. GitHub). See the Nature Research guidelines for submitting code & software for further information.

Data

Policy information about availability of data

All manuscripts must include a data availability statement. This statement should provide the following information, where applicable:

- Accession codes, unique identifiers, or web links for publicly available datasets
- A list of figures that have associated raw data
- A description of any restrictions on data availability

The source data underlying Figs 1b-e and g-m, 2d-g, 3c-p, S1a-f, S3a-d and S4a-d are provided as file entitled Supplementary Data 2. Supplementary Data 2 furthermore contains the full GSEA analysis set of RNAseq data underlying tables S3 (GSEA C5 Biological Processes). The full RNAseq dataset can be accessed from

Gene Expression Omnibus using the accession code GSE154603. All other data are available from authors upon reasonable request.

Field-specific reporting

Please select the one below that is the best fit for your research. If you are not sure, read the appropriate sections before making your selection.

☒ Life sciences ☐ Behavioural & social sciences ☐ Ecological, evolutionary & environmental sciences

For a reference copy of the document with all sections, see nature.com/documents/nr-reporting-summary-flat.pdf

Life sciences study design

All studies must disclose on these points even when the disclosure is negative.

Sample size	Sample size for animal experiments was determined from pilot data which measured flux from hyperpolarized 13C-labelled pyruvate to bicarbonate to be 0.0116 ± 0.0007 s-1 in control animals and 0.006 ± 0.002 s-1 in DOX treated animals. Such a difference would be detectable with 5 rats per group in unpaired studies (G*Power 3.1, $p < 0.05$ with a power of 95%) when PDH flux is the primary endpoint. We therefore used at least 6 animals per group to account for smaller effect size or experiment failure.
Data exclusions	For all CINE MRI and hyperpolarized MRS experiments only paired data from 3 longitudinal experiments (weeks 1, 3 and 6) were used for analysis. If a poor MR spectrum did not allow analysis in for example week 3, week 1 and 6 were also excluded to allow for two-way ANOVA analysis of the whole dataset.
Replication	All experiments were either in vivo or derived from animal tissue. Multiple animals were used per group but ex vivo experiments were only performed once with an appropriate n-number and not replicated in separate identical experiments.
Randomization	Animals were weight-matched into separate cages and the cages then randomized into different treatment groups.
Blinding	Animals were numbered consecutively (DOX01, DOX02, DOX03...) regardless of treatment. Since the same investigator performed the treatments, data acquisition and data analysis proper blinding was not possible.

Reporting for specific materials, systems and methods

We require information from authors about some types of materials, experimental systems and methods used in many studies. Here, indicate whether each material, system or method listed is relevant to your study. If you are not sure if a list item applies to your research, read the appropriate section before selecting a response.

Materials & experimental systems

n/a	Involved in the study	
☒	☐	Antibodies
☒	☐	Eukaryotic cell lines
☒	☐	Palaeontology
☐	☒	Animals and other organisms
☒	☐	Human research participants
☒	☐	Clinical data

Methods

n/a	Involved in the study	
☒	☐	ChIP-seq
☒	☐	Flow cytometry
☒	☐	MRI-based neuroimaging

Animals and other organisms

Policy information about studies involving animals; ARRIVE guidelines recommended for reporting animal research

Laboratory animals	Male Wistar rats, 6-8 weeks of age at start of 6-week protocol
Wild animals	N/A
Field-collected samples	N/A
Ethics oversight	All animal experiments conformed to Home Office Guidance on the Operation of the Animals (Scientific Procedures) Act, 1986 and were approved by a local ethics committee.

Note that full information on the approval of the study protocol must also be provided in the manuscript.
